# Performance of an Ultra-Sensitive Assay Targeting the Circulating Anodic Antigen (CAA) for Detection of *Schistosoma mansoni* Infection in a Low Endemic Area in Brazil

**DOI:** 10.3389/fimmu.2019.00682

**Published:** 2019-04-04

**Authors:** Mariana Silva Sousa, Govert J. van Dam, Marta Cristhiany Cunha Pinheiro, Claudia J. de Dood, Jose Mauro Peralta, Regina Helena Saramago Peralta, Elizabeth de Francesco Daher, Paul L. A. M. Corstjens, Fernando Schemelzer Moraes Bezerra

**Affiliations:** ^1^Medical Sciences Post Graduate Program, Department of Internal Medicine, School of Medicine, Federal University of Ceará, Fortaleza, Brazil; ^2^Parasitology and Mollusks Biology Research Laboratory, Department of Clinical Analysis and Toxicology, Federal University of Ceará, Fortaleza, Brazil; ^3^Department of Parasitology, Leiden University Medical Center, Leiden, Netherlands; ^4^Department of Cell and Chemical Biology, Leiden University Medical Center, Leiden, Netherlands; ^5^Department of Immunology, Institute of Microbiology, Federal University of Rio de Janeiro, Rio de Janeiro, Brazil; ^6^Department of Pathology, Fluminense Federal University, Niterói, Brazil; ^7^Pathology Post Graduate Program, Department of Pathology and Legal Medicine, Federal University of Ceará, Fortaleza, Brazil

**Keywords:** circulating anodic antigen (CAA), diagnosis, up-converting phosphor lateral-flow assay, POC-CCA test, polymerase chain reaction (PCR), *Schistosoma mansoni*, Brazil, low endemic area

## Abstract

Techniques with high sensitivity and specificity are required for an accurate diagnosis in low-transmission settings, where the conventional parasitological methods are insensitive. We determined the accuracy of an up-converting phosphor-lateral flow circulating anodic antigen (UCP-LF CAA) assay in urine and serum for *Schistosoma mansoni* diagnosis in low-prevalence settings in Ceará, Brazil, before and after praziquantel treatment. Clinical samples of a total of 258 individuals were investigated by UCP-LF CAA, point-of-care—circulating cathodic antigen (POC-CCA), soluble worm antigen preparation (SWAP)-ELISA and Kato-Katz (KK); a selection of 128 stools by real-time PCR technique. Three and 6-weeks after treatment, samples were collected and evaluated by detection *Schistosoma* circulating antigens (CAA and CCA). The UCP-LF CAA assays detected 80 positives (31%) with urine and 82 positives (31.8%) with serum. The urine POC-CCA and serum SWAP-ELISA assays detected 30 (11.6%) and 107 (40.7%) positives, respectively. The Kato-Katz technique revealed only 4 positive stool samples (1.6%). Among the 128 individuals with complete data records, 19 cases were identified by PCR (14.8%); Sensitivities and specificities of the UCP-LF CAA assays, determined versus a combined reference standard based on CCA/KK/PCR positivity, ranged from 60–68% to 68–77%, respectively. In addition only for comparative purposes, sensitivities of the different assays were determined vs. a comparative reference based on CAA/KK/PCR positivity, showing the highest sensitivity for the urine CAA assay (80%), followed by the serum CAA (70.9%), SWAP-ELISA (43.6%), PCR (34.5%), POC-CCA (29.1%), whilst triplicate Kato-Katz thick smears had a very low sensitivity (3.6%). CAA concentrations were higher in serum than in urine and were significantly correlated. There was a significant decrease in urine and serum CAA levels 3 and 6-weeks after treatment. The UCP-LF CAA assays revealed 33 and 28 *S. mansoni*-infected patients at the 3- and 6-week post-treatment follow-up, respectively. The UCP-LF CAA assays show high sensitivity for the diagnosis of *S. mansoni* in low-endemicity settings. It detects a considerably higher number of infections than microscopy, POC-CCA or PCR. Also it shows to be very useful for evaluating cure rates after treatment. Hence, the UCP-LF CAA assay is a robust and promising diagnostic approach in low-transmission settings.

## Introduction

In a nation-wide study during a 12-year period, with 12, 491,280 deaths in Brazil, 76,847 deaths (0.62%) were caused by Neglected Tropical Diseases (NTDs). Schistosomiasis was the second cause of death (6,319 deaths; 8.2%), behind only Chagas disease (58,928 deaths; 76.7%) (1). Although mortality has greatly been reduced over the last years, schistosomiasis is still a neglected cause of death in Brazil, with considerable regional differences (2). According to data obtained by the national prevalence survey (2010–2015), an estimated 1.5 million people are infected in Brazil (nearly 1% of the population) (3). A study performed in Pains, state of Minas Gerais, showed that severe clinical forms of schistosomiasis can be present even in such low-endemic areas (4). Another study with a spatiotemporal analysis identified high-risk clusters of deaths, mainly in highly schistosomiasis-endemic areas along the coast of Brazil's Northeast Region (5). In the Ceará state, some isolated foci of intestinal schistosomiasis remain with low parasite burden. According to data from the Brazilian Ministry of Health, the municipality of Capistrano, from 2008 to 2012, was among the five municipalities with the highest prevalence in Ceará state (6).

The diagnosis of *Schistosoma mansoni* infections is routinely performed by the microscopic detection of parasite eggs in the stools (7, 8), using the Kato-Katz, the technique that is still most widely used in epidemiological surveys of intestinal schistosomiasis (9). However, there is a marked decrease in the sensitivity of this method in low-endemicity areas, and thus the development of other tools for diagnosis and surveillance is necessary (10).

Antibody detection is considered highly sensitive and it has been suggested as a complementary tool for the schistosomiasis diagnosis in low endemic areas and/or after large-scale chemotherapy (11–13). However, antibody levels are not associated with the actual worm burden and remain unaffected by treatment of the infection and, consequently, this type of assay has no value when surveying endemic regions (14, 15) as it cannot distinguish between past (previously cured) and new infections. Antibody detection to identify active infections in control programmes will only have value several years after transmission has been interrupted and then still only for newborns from after transmission stop was achieved (16). Antibody detection may also be used to screen travelers with no past infection record (17).

Diagnosis by PCR can be used to detect (egg-derived) DNA of *Schistosoma* in stool (18–21) serum (22, 23), plasma (24), or urine (25–27) and has demonstrated high sensitivity and specificity. A study carried out in Senegal and Kenya raised the PCR as the next reference standard for the diagnosis of *Schistosoma* in stool, particularly useful for *S. mansoni* detection in low transmission areas, post-control settings, and to improves schistosomiasis control programs, epidemiological research, and quality control of microscopy (28).

Alternatively, assays for the detection of *Schistosoma* circulating antigens (adult worm gut-associated antigens) have been described. The circulating cathodic antigen (CCA) and the circulating anodic antigen (CAA) are both applied to diagnose active infections and indicated to be a useful approach also in evaluation drug efficiency, and the assessment of cure (29–32).

Various studies have been conducted in endemic areas of the African, Asian and South American continents to determine the efficacy and field applicability of the commercially available rapid Point-of-Care test (POC-CCA) for diagnosis of intestinal schistosomiasis (i.e., *S. mansoni* infection). A summary of 5 countries' evaluations of the POC-CCA test, funded by the Schistosomiasis Consortium for Operational Research and Evaluation (SCORE), demonstrated that this method is recommended as a mapping tool for the detection of *S. mansoni* in endemic areas (33–35). Up to now only a few studies have been published showing the results of POC-CCA trials in Brazil. Overall, they have shown that the POC-CCA is capable of detecting many additional positive cases compared to Kato-Katz thick smears, in particular in low endemic areas, but they also reported that the true significance of the trace readings remains unclear (4, 36–38). A systematic review about Kato-Katz vs. POC-CCA for *Schistosoma mansoni* indicated that below 50% of prevalence, the POC-CCA test is more sensitive than the Kato-Katz, but that further research in low- prevalence areas is needed to understand how best to manage places that have very low or no prevalence by Kato-Katz, but significant prevalence using POC-CCA or others new assays (39).

In order to continue the development of rapid tests for the schistosomiasis diagnosis that may be employed in future as Point-of-Care applications, and also to further improve robustness, the ultra-sensitive up-converting phosphor (UCP) technology was introduced in combination with a lateral flow-based platform for the CAA detection in serum (UCP-LF CAA assay). This assay achieved an analytical sensitivity below 1 pg/mL, about 10-fold better than that of the CAA-ELISA (29, 40–42). The concentration of 1 pg/mL in serum is expected to allow identification of single worm infections, since *in vitro* worm culture studies and studies with experimentally infected baboons indicate steady state serum CAA levels of approximately 5 pg/mL (43, 44). Corstjens et al. (29) have shown that the UCP-LF format was also successfully applied for detection of CAA levels in urine and after a concentration step using disposable centrifugal concentration devices shows almost absolute accuracy to diagnose schistosomiasis. Whereas, the POC-CCA test is most applied to demonstrate *S. mansoni* infection, the UCP-LF CAA test is a genus-specific test which can be applied to demonstrate infections with any *Schistosoma* species, including veterinarian (29, 45). Variants of the assay format were evaluated with banked urine samples from highly endemic settings in the Philippines for *S. japonicum* and in Cambodia and Lao People's democratic Republic for *S. mekongi* (35, 46). The test has also been successfully applied in low transmission settings in the People's Republic of China, in Tanzania and Burundi for the diagnosis of, respectively, *S. japonicum* (47), *S. haematobium* (48), and *S. mansoni* infections (49).

Recently, Colley et al. (16) reported the crucial necessity for continued evaluation of the assays involved and for new guidelines based on the use of the more sensitive assays for those NTD programs that wish to move forward to strategies designed for elimination. Here, the current study focuses on a full evaluation of the UCP-CAA lateral flow test (UCP-CAA LF) in a low endemic area in Brazil, comparing the results with several diagnostic methods including Kato-Katz, POC-CCA, PCR, and antibody detection before treatment (baseline). Additionally, the UCP-CAA LF assays and POC-CCA test were utilized for determining the efficacy of treatment by follow-up testing of urine and serum samples 3 and 6 weeks post-treatment. In an accompanying previous article (36), the performance of the POC-CCA was compared with Kato-Katz technique in the same community, resulting in a 7-fold increase in prevalence of *S. mansoni* and also it was compared with antibody detection.

## Materials and Methods

### Ethics Statement, Recruitment and Treatment

The study protocol was approved by the Universidade Federal do Ceara ethical committee (application no. 480.719). It was conducted by the Resolution No. 466/12 of the Brazilian Health Council. District health authorities and all community were informed about the purpose, procedures, and potential risks and benefits of the study. Before enrollment, informed consent was obtained in writing or by fingerprint (in cases of illiteracy). Parents/ legal guardians provided informed consent for their children to participate. Participation was voluntary and anyone could withdraw at any time without further obligation. All samples obtained in the study were coded and treated confidentially.

Treatment with praziquantel (PZQ) (Farmanguinhos, Ministry of Health, Brazil) was offered to all individuals free of charge regardless of infection status. The collective treatment was applied because this study is part of a larger project, whose objective is to interrupt the transmission of schistosomiasis in this area. It was done with a single dose of 60 mg/kg for children (≤15 years old) and 50 mg/kg for adults, as recommended by the Brazilian Ministry of Health. All treatment was supervised and confirmed by a doctor and a nurse from district. The younger children participating were treated with crushed PZQ tablets mixed with some of juice and the efficacy and safety of this intervention have been described elsewhere (50).

### Study Area and Population

The study pursued a 3- and 6-week longitudinal design with a treatment intervention and was conducted between April and October 2013 in the community of Bananeiras, a rural locality belonging to the Capistrano municipality, in Ceará state, Northeast of Brazil (geographical co-ordinates 4° 28′ 20″S latitude, 38° 54′ 14″W longitude). Capistrano extends for 222.6 km^2^ and is located at 155 meters of altitude in relation to the level of the sea, about 93 km south of Fortaleza, the capital of Ceará. The region is endemic for *S. mansoni* and this has been documented since the early surveys in Ceara State by the control program in 1976. Situated in a region of semi-arid climate, the river which passing through the village (Aracoiaba River) remains dry most of the year, with the flood season between the months of December and March. Our door-to-door census conducted in March 2013 revealed 297 people aged 2 years or above in Bananeiras community. This area was chosen because the positivity rate of schistosomiasis reported in 2010 was 1.6% (3 positives/ *n* = 188) and there has been no specific treatment for schistosomiasis in the past 2 years.

The 3- and 6-week time-points post-treatment were chosen for optimal reduction of test parameters and before reinfection can be recorded (51, 52).

### Sample Collection

Infection with *S. mansoni* was assessed during a baseline cross-sectional survey and again 3 and 6 weeks after PZQ administration. Individuals (aged 2 years or above) with informed consent and no recent treatment for schistosomiasis (at least within in the past 2 years) were invited for stool, urine and blood sample collection at baseline.

At baseline, one day before the collect-day two plastic containers labeled with unique identification numbers (IDs) (stool and urine containers) were delivered to each study participant/mothers or guardians, at this time also venous blood samples were taken. Individual serum samples were obtained after centrifugation of coagulated blood samples at 3,000 g for 5 min and these were stored frozen at −20°C. On the following day, the community was invited to return the containers filled with a fresh morning urine sample and a stool sample to the fieldworkers stationed at Bananeiras Health Center, where the samples were processed the same day. Small aliquots of urine (5 mL) and of serum (2 mL) were frozen and kept at −20°C in Parasitology and Mollusks Biology research laboratory at Universidade Federal do Ceara in Brazil prior to transfer to the Department of Parasitology, Leiden University Medical Center (LUMC) in the Netherlands for circulating anodic antigen testing. Three and six weeks after PZQ administration, urine, and serum samples were collected again, using the same procedures.

## Laboratory Procedures

### Microscopy

Three Kato-Katz thick smears slides per stool sample, using 41.7 mg templates, were prepared and quantitatively examined for *S. mansoni* (53). Each slide was read by two trained microscopists and any discrepancies resolved before results were recorded as egg counts per slide and multiplied by a factor of 24 to convert it into eggs per gram of stool (EPG). For quality control, 10% of the Kato-Katz thick smears were re-examined by a senior technician from Rene Rachou Research Center, Oswaldo Cruz Foundation, Minas Gerais, Brazil.

### UCP-LF CAA Assays

Additionally, all urine and serum samples were frozen and transported on dry ice to LUMC in Leiden, where they were stored at −20°C. Overall, urine and serum samples from 128 patients were examined by an upconverted phosphor lateral flow (UCP-LF) assay for schistosome CAA before and 3 and 6 weeks after PZQ administration. Urine samples were investigated by a highly sensitive concentration-based assay (UCAA2000 format, as described elsewhere (29). Briefly, 2 mL urine samples were diluted with an equal volume of 4% (w/v) tri-chloro-acetic acid (TCA), centrifuged and then the clear supernatants were reduced to amounts of 20–30 μL using an Amicon Ultra-4 device (EMD Millipore; Billerica, MA, USA). After incubation with the UCP-antibody conjugate solution, strips were added and the samples allowed to run as described previously (29). Following overnight drying, the strips were scanned for bound UCP, using a Packard FluoroCount microtiter plate reader adapted with an IR laser (980 nm) modified to scan LF strips (54).

The serum samples were examined in a similar way using the UCP-LF assay with a concentration-based assay utilizing 500 μL of serum (SCAA500). It followed an analogous procedure to the UCAA2000 method, with 500 μL serum TCA supernatant being concentrated over an Amicon Ultra-0.5 device to 20 μL.

Test line signals (T) were normalized to flow control signals (FC) of the individual LF strips and results expressed as a ratio value (T/FC) (29, 55). Standard curves of CAA spike in negative urine/serum were used to quantify CAA levels in the clinical samples (30). In more detail, dilution series of partly purified antigen, i.e., the TCA-soluble fraction of *S. mansoni* adult worm antigen (AWA) containing 3% CAA (AWA-TCA) in negative urine/serum, were assayed with each set of clinical samples (50 urine/100 serum) and the respective standard curves were included for calculation of the CAA concentration (pg/mL urine/serum) in the original sample as described elsewhere (40). The assay cut-offs were decided in accordance with Corstjens et al. (29) using 0.1 pg/mL urine and 1 pg/mL serum as the lower limits of detection (LOD), and 0.05 pg/mL urine and 0.5 pg/mL serum as the LOD if the assay would have been performed with multiple samples under ideal laboratory conditions. The region between the two LODs was designated as “indecisive,” indicating that samples are suspected to be positive but to truly ascertain the positive or negative score, the samples would need to be retested, preferable at higher sample volume.

### Circulating Cathodic Antigen (CCA) Test

Urine samples were tested using a commercially available POC-CCA cassette test (Batch no. 33,827, Rapid Medical Diagnostics, Pretoria, South Africa) performed at ambient temperature according to the manufacturer's instructions on the day of sample collection. Briefly, one drop of urine was added to the well of the testing cassette and allowed to absorb. Once fully absorbed, one drop of buffer (provided with the CCA test kits) was added. The test results were read 20 min after adding the buffer. Valid tests were scored as negative, trace or positive, according to the visibility of the color reaction and the manufacturer's instructions. All tests were read independently by two blinded investigators and in case of discordant results discussed with a third independent investigator until agreement was reached. Urine samples collected at all time points in the study were subjected as described above.

### DNA Isolation and Real-Time PCR

For specific real-time PCR, total genomic DNA was extracted from stool specimens using the NucleoSpin® Soil (MACHEREY- NAGEL, Düren, Germany) according to the manufacturer's instructions. Stool suspensions from each clinical specimen were prepared from 1 g of unpreserved feces in deionized water. Samples were disrupted in the Mini-Beadbeater-16 cell disruptor instrument (BioSpec Products, Bartlesville, OK, USA) for 1 min. DNA was stored at −20°C until used in real-time PCRs. For the detection of *S. mansoni*—specific egg DNA, the primers SMCYT748F and SMCYT847R were selected to amplify a fragment of 99 bp, which is detected by the double-labeled probe, SMCYT785T-FAM (19). For the amplification, 5 μL of DNA extracted from stool specimens was used as a template (sample, positive and negative DNA controls) with 11 μL of Platinum Quantitative PCR Supermix-UDG with ROX (Invitrogen, Carlsbad, CA, USA) added with 0.55 μL of each primer, 1.1 μL of the probe, and 1.8 μL of deionized water, resulting in a total reaction volume of 20 μL. Primers and probe were used at 0.19 μM and 0.139 μM concentrations, respectively. Each unknown sample was diluted 1:2 and 1:20, and both dilutions were used in the assay. Amplification consisted of 2 min at 95°C followed by 40 cycles of 15 s at 95°C and 30 s at 60°C. Amplification, detection and data analysis were performed with the Applied Biosystems 7500 Fast Real Time PCR System (Applied Biosystems, Foster city, CA, USA). DNA amplification was positive when Ct values were < 38. We choose the samples from the 128 followed-up individuals to the partial analysis by PCR at baseline.

### Serology

The ELISA assay was performed for the detection of IgG antibody against soluble worm antigen preparation (ELISA-SWAP) according to the methodology described by Grenfell et al. (13).

### Statistical Analysis

Data were entered in a spreadsheet (Excel 2007^TM^). Statistical analysis was done using GraphPad Prism 5 (GraphPad Software Inc.; California, USA) and SPSS version 20 (IBM Corp.; Armonk, USA). Non-parametric statistics were used for correlation between test results, to assess the CAA levels before and after drug treatment and the correlation and differences between urine and serum CAA concentrations. Agreement between binary variables was established by determining the kappa statistics (k). Specificity and sensitivity were calculated and used for indication of the performances of the UCP-LF CAA assays and of the SWAP-ELISA against a combined reference standard constructed by Kato-Katz and/or PCR and/or POC-CCA (considering “trace” readings as negative) results; On the other hand, specificity, sensitivity and positive and negative predictive values of the diagnostic tests were calculated following an approach described previously (47, 48), but only for comparative reasons. For this purpose, a comparative reference was constructed, defined as being positive if a sample from an individual presented with *Schistosoma mansoni* eggs at least once out of three thick smears (Kato-Katz) and/or found positive in the PCR and/or the UCP-LF CAA assay (urine or serum), considering “indecisive” readings as negative. This approach implied that, by definition, the specificities and consequently the positive predictive values of the individual tests were taken as 100%. Due to the very high specificities of the circulating antigen assays used this was considered valid (56). In addition, an “indecisive” or “trace” category was defined and analyzed separately, both for the CAA assays and the POC-CCA results, following the approach described by Coulibaly et al. (57), Van Dam et al. (35), and Vonghachack et al. (46). A two-way analysis of CAA data in urine/serum with “indecisive” results considered infection-negative (UCAA-/SCAA-) and “indecisive” results considered infection-positive (UCAA+/SCAA+) were performed. Moreover, this was not a crossectional study as well as that the parameters are influence by prevalence. Cure rates were calculated from individuals for whom a complete UCAA2000 or SCAA500 results was obtained at all time points in the study. Cure rates were calculated as the proportion of patients positive by UCAA2000 or SCAA500 before treatment who became negative 3 or 6 weeks after treatment. A 5% level of significance was adopted for all inferential procedures.

## Results

### Adherence and Population Characteristics

**Figure 1:**
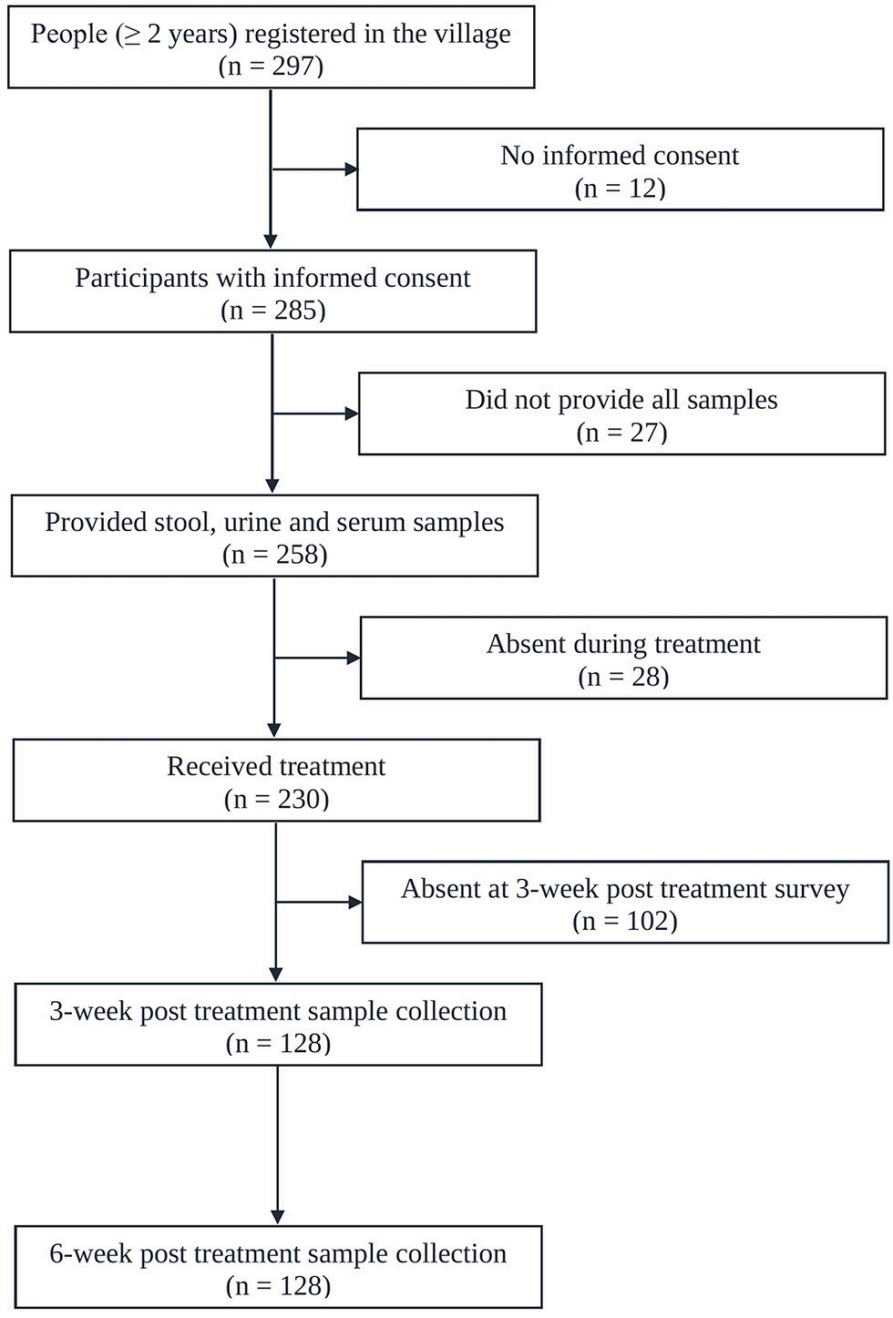
Flowchart showing study participation. Flowchart detailing the study participation and adherence of people for submitting samples for the diagnosis of *S. mansoni* infection before and after administration of praziquantel in Bananeiras village, Ceara, Brazil, between April and October 2013.

Figure 1 shows the adherence of people to the study protocol. Our population census revealed 297 people aged 2 years or above, all of whom were invited to participate. Twelve individuals had no written informed consent. From the 285 individuals participating at the baseline cross-sectional survey, twenty-seven were excluded due to incomplete data (e.g., insufficiently large stool sample for triplicate Kato-Katz thick smears). The 258 remaining people provided stool, urine and blood samples for a suite of diagnostic tests for detection of *S. mansoni* (triplicate Kato-Katz thick smears, SWAP-ELISA, one POC-CCA test, and UCP-LF CAA assays). There were 140 female (54.3%) and 118 male with a median age of 30 years (range: 2 to 87 years). Among them 230 received PZQ treatment. Three and six weeks post-treatment, except for a few losses in certain tests, we were able to re-examine urine and serum sample from 128 patients (re-examined with one POC-CCA test and UCP-LF CAA assays). Among them 40.6% (*n* = 52) were male and the median age of the cohort was 19 years (age range 2–87 years). The molecular diagnostic at baseline and consequently the accuracy analysis was focused on this cohort of people.

### Individual Test Results and *S. mansoni* Prevalences Before Treatment According to Diagnostic Approach

Only four out of the 258 stool samples at baseline were found positive by Kato-Katz thick smears, owing to a prevalence of 1.6%. The prevalence for a single, duplicate and triplicate smear readings were 0.8, 1.2, and 1.6%, respectively. All the positive ones were classified as having a very low intensity infection (arithmetic means fecal egg counts equal to 8 EPG). Meantime, we found 80 urine CAA positives (31.0%), 82 serum CAA positives (31.8%), and 10 POC-CCA positives (3.9%). Additionally, in the POC-CCA test, 20 cases were scored trace (7.7%) whereas in the urine CAA assay, 13 cases were scored indecisive (5.0%) and 14 cases (5.4%) in the serum CAA. SWAP-ELISA detected 105 (40.7%) positive cases. Table 1 shows those positive for *S. mansoni*, as assessed by different diagnostic approaches. The 95% confidence intervals (95% CI) for prevalence are shown. In the PCR sampling, we found 19 positives (14.8%; *n* = 128). There was no association between infection and gender (*p* = 0.46). Table 2 shows the Kato-Katz, the urine and serum CAA and the PCR positive cases, according to different age-groups. The highest positivity occurred in adults regardless of the diagnostic approach, with a maximum between 20 and 39 years-old.

**Table 1:** Baseline prevalence of *S. mansoni* according to different diagnostic approaches (*n* = 258).

**Diagnostic**	**Infected (N^**°**^.)**	**% positive (95% CI)**
Kato-Katz	4	1.6 (0.0–3.1)
UCAA2000−	80	31 (25–37)
UCAA2000+	93	36 (30–42)
SCAA500−	82	32 (26–38)
SCAA500+	96	37 (31–43)
POC-CCA (t−)	10	3.9 (1.5–6.2)
POC-CCA (t+)	30	12 (7.7–16)
SWAP-ELISA	105	41 (35-47)

*UCP-LF CAA: up-converting phosphor—lateral flow assay detecting circulating anodic antigen; UCAA 2000–: UCP-LF CAA prepared with 2 mL of urine, indecisive results were considered as negative; UCAA 2000+: UCP-LF CAA prepared with 2 mL of urine, indecisive results were considered as positive; SCAA500–: UCP-LF CAA prepared with 0.5 mL of serum, indecisive results were considered as negative; SCAA500+: UCP-LF CAA prepared with 0.5 mL of serum, indecisive results were considered as positive; POC-CCA: rapid urine based point-of-care circulating cathodic antigen test; t–, trace negative; t+, trace positive; CI, confidence interval*.

**Table 2:** Number of positive cases for the respective age group, according to different diagnostic approaches.

**Age group**	**Number tested**	**Positives (*****n*****/%)**			Tested^*^	Positives (*n*/%) PCR^*^
		**Microscopy**	**UCAA–**	**SCAA–**		
2–9	32	0	3/9.4	4/13	26	2/7.7
10–19	58	0	12/20	12/21	39	3/7.7
20–29	38	0	16/42	16/42	17	5/29
30–39	43	3/7	20/47	20/47	20	6/30
40–49	35	1/2.8	11/31	14/40	14	1/7.1
50–59	23	0	9/39	6/26	5	2/40
>60	29	0	9/31	10/35	7	0
Total	258	4/1.6	80/31	82/32	128	19/15

UCAA–: UCP-LF CAA assay detecting circulating anodic antigen in urine, indecisive results were considered as negative; SCAA–: UCP-LF CAA assay detecting circulating anodic antigen in serum, indecisive results were considered as negative;

* *PCR was performed by sampling (n = 128)*.

## Diagnosis Accuracy of Different Tests Before Treatment

### Sensitivity and Specificity of Diagnostic Techniques Using a Combined Reference Standard

Table 3 shows the numbers of positive, negative, indecisive and trace results for each diagnostic method combination in 6-cell and nine-cell-matrixes. Of the four egg-positive patients, only one was positive in the POC-CCA and two others were positive both in the UCP-LF CAA assays and in the PCR. Noteworthy, one out of the four egg-positive cases, 11 (57.9%) of the PCR positives and 51 (53.1%) of the CAA positives were antibody negative.

**Table 3:** Agreement between the different diagnostic approaches.

	**Positive**	**Trace/indecisive**	**Negative**	**Total**	K^*^	***P*-value**
**UCAA2000**	**POC-CCA**					
Positive	7	11	62	80		
Indecisive	0	0	13	13	0.09	0.007
Negative	3	9	153	165		
Total	10	20	228	258		
	**SCAA500**					
Positive	66	6	8	80		
Indecisive	2	1	10	13	0.73	0.000
Negative	14	7	144	165		
Total	82	14	162	258		
**PCR**	**UCAA2000**					
Positive	14	1	4	19		
Negative	30	5	74	109	0.30	0.000
Total	44	6	78	128		
	**SCAA500**					
Positive	13	2	4	19		
Negative	26	5	78	109	0.31	0.000
Total	39	7	82	128		
	**POC-CCA**					
Positive	3	7	9	19		
Negative	5	9	95	109	0.15	0.063
Total	8	16	104	128		

*Six-cell and nine-cell-matrixes showing the agreement of the number of positive, negative, indecisive, and trace results of the up-converting phosphor—lateral flow assay detecting circulating anodic antigen in urine (UCAA2000) and in serum (SCAA500), the POC-CCA test and the PCR for the diagnosis of S. mansoni in samples from Brazil*.

* *Kappa indexes: trace and indecisive results were considered as negative*.

The sensitivities and specificities of the SWAP-ELISA and of the UCP-LF CAA assays, including the separate analysis with indecisive results being considered as negative or positive, versus the combined reference standard (combination of the triplicate Kato-Katz thick smears, PCR and POC-CCA test with trace results considered as negative) are summarized in Table 4. A total of 128 cases had data from all assays with 25 positive by Kato-Katz and/or PCR and/or CCA. The sensitivities of the UCP-LF CAA assays were similar in both clinical samples (range 60–68%) and higher than SWAP-ELISA (40%). The specificities of all these assays were similar (range 67–77%).

**Table 4:** Number of positive and negative results of the up-converting phosphor–lateral flow assay detecting circulating anodic antigen in urine (UCAA2000) and in serum (SCAA500), and the SWAP-ELISA for the diagnosis of *S. mansoni* against a combined reference standard of infection-positivity by either egg (Kato-Katz) and/or PCR and/or POC-CCA test in samples from Brazil.

	KatoKatz and/or PCR and/or POC-CCA results as combined standard^*^		
	**Positive**	**Negative**	**Total**
**UCAA2000-**			
Positive	16	28	44
Negative	9	75	84
Total	25	103	128
Sensitivity	64%	Specificity	73%
**UCAA2000+**			
Positive	17	33	50
Negative	8	70	78
Total	25	103	128
Sensitivity	68%	Specificity	68%
**SCAA500-**			
Positive	15	24	39
Negative	10	79	89
Total	25	103	128
Sensitivity	60%	Specificity	77%
**SCAA500+**			
Positive	17	29	46
Negative	8	74	82
Total	25	103	128
Sensitivity	68%	Specificity	72%
**SWAP-ELISA**			
Positive	10	34	44
Negative	15	69	84
Total	25	103	128
Sensitivity	40%	Specificity	67%

* *i.e., combined reference standard, assuming 100% specificity of the egg detection (Kato-Katz) and/or PCR and/or CCA results, considering “trace” readings as negative (n = 25 positives). UCAA 2000-: UCP-LF CAA prepared with 2 mL of urine, indecisive results were considered as negative; UCAA 2000+: UCP-LF CAA prepared with 2 mL of urine, indecisive results were considered as positive; SCAA500–: UCP-LF CAA prepared with 0.5 mL of serum, indecisive results were considered as negative; SCAA500+: UCP-LF CAA prepared with 0.5 mL of serum, indecisive results were considered as positive*.

The sensitivities, specificities and the positive and negative predictive values of the different assays, including the separate analysis with trace/indecisive results being considered as negative or positive, vs. a comparative reference (combination of the triplicate Kato-Katz thick smears, PCR and UCP-LF CAA assay (urine and serum) with indecisive results considered as negative) are summarized in Table 5. A total of 128 cases had data from all assays with 55 positive by Kato-Katz, PCR and/or urine or serum CAA. The UCAA2000+ had the highest sensitivity of 83.6%, followed by UCAA2000- and SCAA500+ both with a sensitivity of 80.0%. The sensitivities of UCP-LF CAA assays, SWAP-ELISA (43.6%) and PCR (34.5%) or even the single CCA test (*t*+ = 29.1%; *t*– = 10.9%) were considerably higher than the stool examination with three Kato-Katz thick smears, which showed very low sensitivity (3.6%). Also the UCAA2000+ had the highest NPV of 88.5%, followed by the UCAA2000- with a NPV of 86.9%. Sensitivity improvements were obtained by the combination of urine and serum CAA findings (87.3%; 89.1%) and by the combination of CAA assays and CCA test findings (89.1%; 94.5%).

**Table 5:** Diagnostic characteristics of various assays used for the diagnosis of *S. mansoni* against a comparative reference of infection-positivity by either egg (Kato-Katz) and/or PCR and/or UCP-LF CAA assay^a^, in samples from Brazil (*n* = 55 positives).

**Comparative reference^a^**	**Sensitivity (95% CI)**	**Specificity (95% CI)**	**PPV (95% CI)**	**NPV (95% CI)**
Kato-Katz	3.6 (0.0–8.6)	b.d.^a^	b.d.^a^	58 (49–67)
PCR	35 (22–47)	b.d.	b.d.	67 (58–76)
UCAA2000–	80 (69–91)	b.d.	b.d.	87 (80–94)
UCAA2000+	84 (74–93)	95 (89–100)	92 (85–100)	89 (81–96)
SCAA500–	71 (59–83)	b.d.	b.d.	82 (74–90)
SCAA500+	80 (69–91)	97 (94–100)	96 (90–100)	87 (79–94)
UCAA2000– and SCAA500–^b^	87 (79–96)	b.d.	b.d.	91 (85–97)
UCAA2000+ and SCAA500+^b^	89 (81–97)	92 (86–98)	89 (81–97)	92 (86–98)
POC-CCA (t–)	11 (2.7–19)	97 (94–100)	75 (45–100)	59 (50–68)
POC-CCA (t+)	29 (17–41)	89 (82–96)	67 (48–86)	63 (53–72)
UCAA2000–, SCAA500–, and POC–CCA (t–)^c^	89 (81–97)	97 (94–100)	96 (91–100)	92 (86–98)
UCAA2000+, SCAA500+, and POC CCA (t+)^c^	95 (89–100)	81 (72–90)	79 (69–89)	95 (90–100)
SWAP-ELISA	44 (31–57)	73 (62–83)	55 (40–69)	63 (53–73)

a *i.e., comparative reference, assuming 100% specificity of the egg detection (Kato-Katz), PCR and CAA results, considering “indecisive” readings as negative. Therefore, by definition (b.d.) specificity and positive predictive values are 100%*.

b *CAA in urine and/or serum*.

c *Combined of CAA assays and CCA test findings with trace/indecisive results considered as negative or positive. UCP-LF CAA: up-converting phosphor—lateral flow assay detecting circulating anodic antigen; UCAA 2000–: UCP-LF CAA prepared with 2 mL of urine, indecisive results were considered as negative; UCAA 2000+: UCP-LF CAA prepared with 2 mL of urine, indecisive results were considered as positive; SCAA500–: UCP-LF CAA prepared with 0.5 mL of serum, indecisive results were considered as negative; SCAA500+: UCP-LF CAA prepared with 0.5 mL of serum, indecisive results were considered as positive; POC-CCA: rapid urine based point-of-care circulating cathodic antigen test; CI, confidence interval; PPV, Positive Predictive Value; NPV, Negative Predictive Value; t–, trace negative; t+, trace positive*.

### Post-treatment Readings and Efficacy Evaluation

We found 34 (26.8%) and 35 (27.3%) urine and 13 (10.9%) and 11 (8.9%) serum CAA positives at 3 and 6 weeks after treatment, respectively. Additionally, in the urine CAA assay, 6 (4.7%) and 30 (23.4%) cases were scored indecisive at 3 and 6 weeks after treatment and 6 (5.0%) and 7 (5.6%), in the serum CAA assay, respectively (Table 6). Meanwhile, we found 2 CCA positives (1.6%) and 3 (2.3%) trace results in the POC-CCA test at 3 weeks after treatment. At the 6-week post-treatment evaluation, all patients were CCA negatives.

**Table 6:** Number of urine and serum baseline CAA positive cases, compared to 3 and 6 weeks after treatment with praziquantel.

		**3 weeks after treatment**			**6 weeks after treatment**		
**Baseline**	**Total**	**Positive**	**Indecisive**	**Negative**	**Positive**	**Indecisive**	**Negative**
**UCAA2000**^**a**^							
Positive	44	32	4	8	27	8	9
Indecisive	6	0	0	6	1	0	5
Negative	78	2	2	73	7	22	49
Total	128	34	6	87	35	30	63
Positivity	34%	27%			27%		
**SCAA500**^**b**^							
Positive	39	12	6	18	10	5	21
Indecisive	7	1	0	6	0	0	7
Negative	82	0	0	76	1	2	78
Total	128	13	6	100	11	7	106
Positivity	31%	11%			8.9%		

a *One patient have no UCAA2000 result at 3 weeks after treatment*.

b *Nine and four patients have no SCAA500 results at 3 and 6-weeks after treatment, respectively*.

Among the 44 patients who were UCAA2000- positives at baseline, 12 (27.3%) became UCAA2000- negatives on repeat testing performed 3 weeks post-treatment. Seven (21.9%) of the remaining UCAA2000- positives (*n* = 32) became negative based on follow-up UCAA2000- testing at 6 weeks post-treatment. Meanwhile, among the 34 patients who were SCAA500- positives at baseline, 23 (67.6%) became SCAA500- negatives on repeat testing performed 3 weeks post-treatment. Four (36.4%) of the remaining SCAA500- positives (*n* = 11) became negative based on follow-up SCAA500- testing at 6 weeks post-treatment.

The UCP-LF CAA assays (serum and urine), considering “indecisive” as negative results, revealed 33 and 28 *S. mansoni*-infected patients at the 3- and 6-week post-treatment follow-up, respectively. The respective Cure Rates (CRs) were 31.2 and 41.7%. The CRs are showed in more details according with the clinical sample applied (UCAA2000/SCAA500), considering “indecisive” as positive or negative results in the Table 7.

**Table 7:** Diagnosis of *S. mansoni* infection by UCAA2000 and SCAA500 methods, 3–6-weeks after treatment.

	**Pre-treatment** ***n*** **(%)**			
	**Indecisive pos**		**Indecisive neg**	
	**Positive**	**Negative**	**Positive**	**Negative**
**3-WEEK POST- TREATMENT**				
**UCAA2000**				
Positive	36 (72)	4 (5.2)	32 (73)	2 (2.4)
Negative	14 (28)^*^	73 (95)	12 (27)^*^	81 (98)
**SCAA500**				
Positive	19 (44)	0	12 (33)	1 (1.2)
Negative	24 (56)^*^	76 (100)	24 (67)^*^	82 (99)
**6-WEEK POST-TREATMENT**				
**UCAA2000**				
Positive	36 (72)	29 (37)	27 (61)	8 (9.5)
Negative	14 (28)^*^	49 (63)	17 (39)^*^	76 (90.5)
**SCAA500**				
Positive	15 (35)	3 (3.7)	10 (28)	1 (1.1)
Negative	28 (65)^*^	78 (96)	26 (72)^*^	87 (99)

* *Percentage cure as determined considering indecisive result as positive or negative*.

### Correlation of Urine and Serum CAA Levels

**Figure 2:**
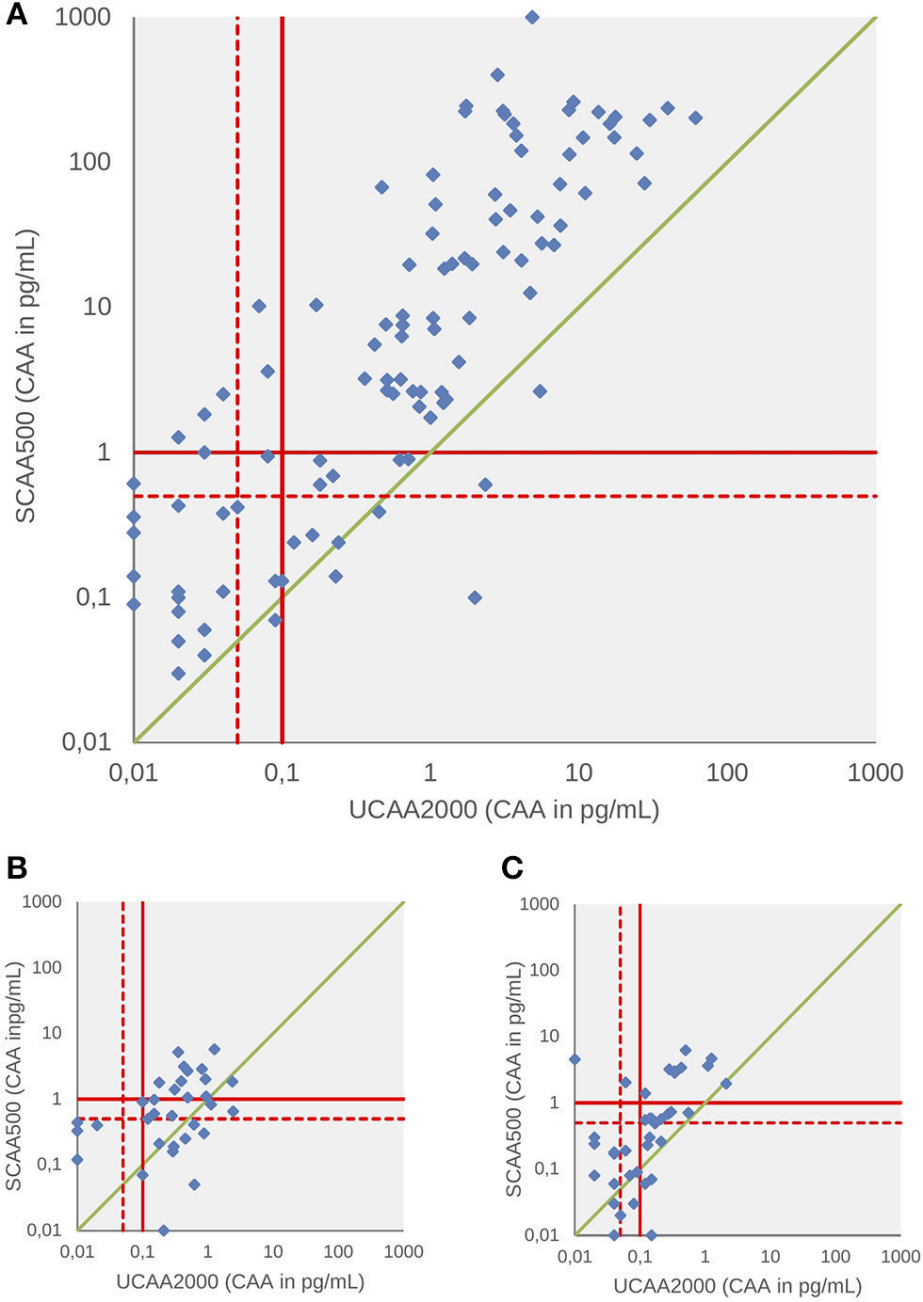
Scattergram of urine and serum CAA concentrations as determined by the UCP-LF CAA assay at baseline **(A)**, 3 weeks **(B)**, and 6 weeks after drug treatment **(C)**. The solid red lines indicate high-specificity cut-off levels, while the dotted red lines indicate lower specificity levels. Samples that have concentrations in the in-between region would be classified as “indecisive.” The solid green diagonal line represents where CAA concentrations in serum and urine would be equal, indicating that most serum CAA concentrations are higher than urine CAA.

A total of 258,118 and 124 patients had both serum and urine samples subjected to CAA testing at baseline, 3 and 6-weeks after treatment, respectively. We found significant correlations between the concentrations in all three points: baseline (panel A, Spearman's rho 0.66, *p* < 0.001), 3-weeks (panel B, Spearman's rho 0.60, *p* < 0.001) and 6-weeks after treatment (panel C, Spearman's rho 0.45, *p* < 0.001) (Figure 2). Serum CAA concentrations were significantly higher than those in urine [Baseline—Serum CAA (24 ± 83), Urine CAA (1.7 ± 5.9), *p* < 0.001; 6 weeks after PZQ—Serum CAA (0.36 ± 1.03), Urine CAA (0.11 ± 0.24), *p* < 0.001], except in 3-week analysis after treatment [Serum CAA (0.35 ± 0.90), Urine CAA (0.18 ± 0.45), *p* = 0.45] (Mann-Whitney test).

### Praziquantel Efficiency by Changes in the CAA Levels

**Figure 3:**
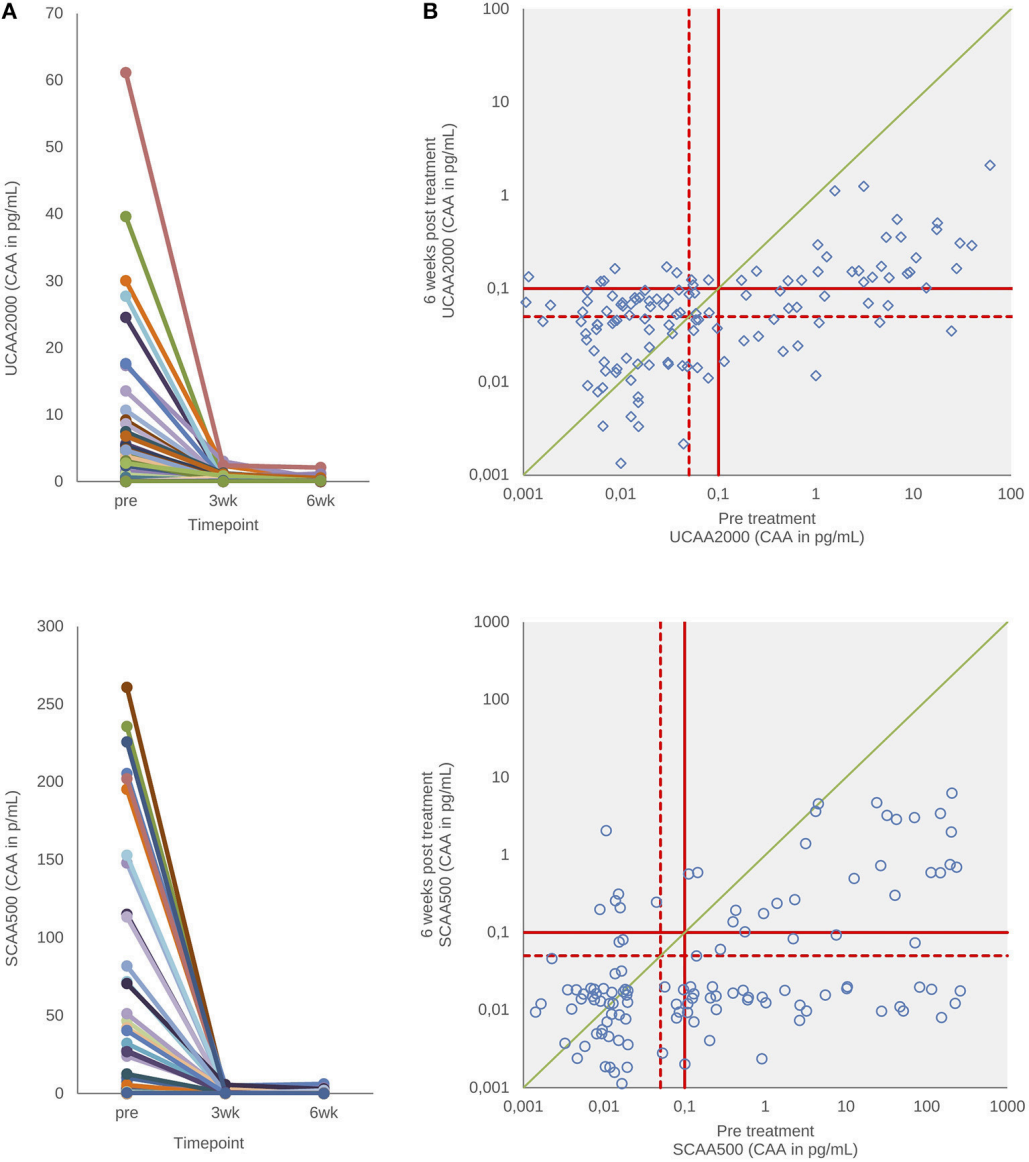
CAA levels before and after drug treatment. **(A)** The decrease in urine and serum CAA concentrations showing the respective values before and 3–6 weeks after treatment with praziquantel. **(B)** Scatter plot of the urine and serum CAA concentrations as determined before and 6 weeks after treatment. The solid red lines indicate high-specificity cut-off levels, while the dotted red lines indicate lower specificity levels. Samples that have concentrations in the in-between region would be classified as “indecisive.” The solid green diagonal line in **(B)** indicates the “no change in CAA concentration” position; samples with values below this line indicate a decrease of the CAA concentration 6 weeks after treatment.

Figure 3 shows urine and serum CAA levels before and 3–6 weeks after administration of the drug praziquantel. Panel A shows decrease of urine and serum CAA levels 3 (*p* < 0.001) and 6 weeks (*p* < 0.001) after treatment. There was no difference between 3 and 6 weeks in both clinical samples (*p* = 0.78, urine; *p* = 0.93, serum) (Wilcoxon test). The urine and serum CAA concentrations of 27 and 10 patients, respectively, remained above the high specificity cut-off threshold at 6 weeks after treatment (panel B).

### Correlation of *S. mansoni* Urine CAA Levels With POC-CCA Intensity Scores

**Figure 4:**
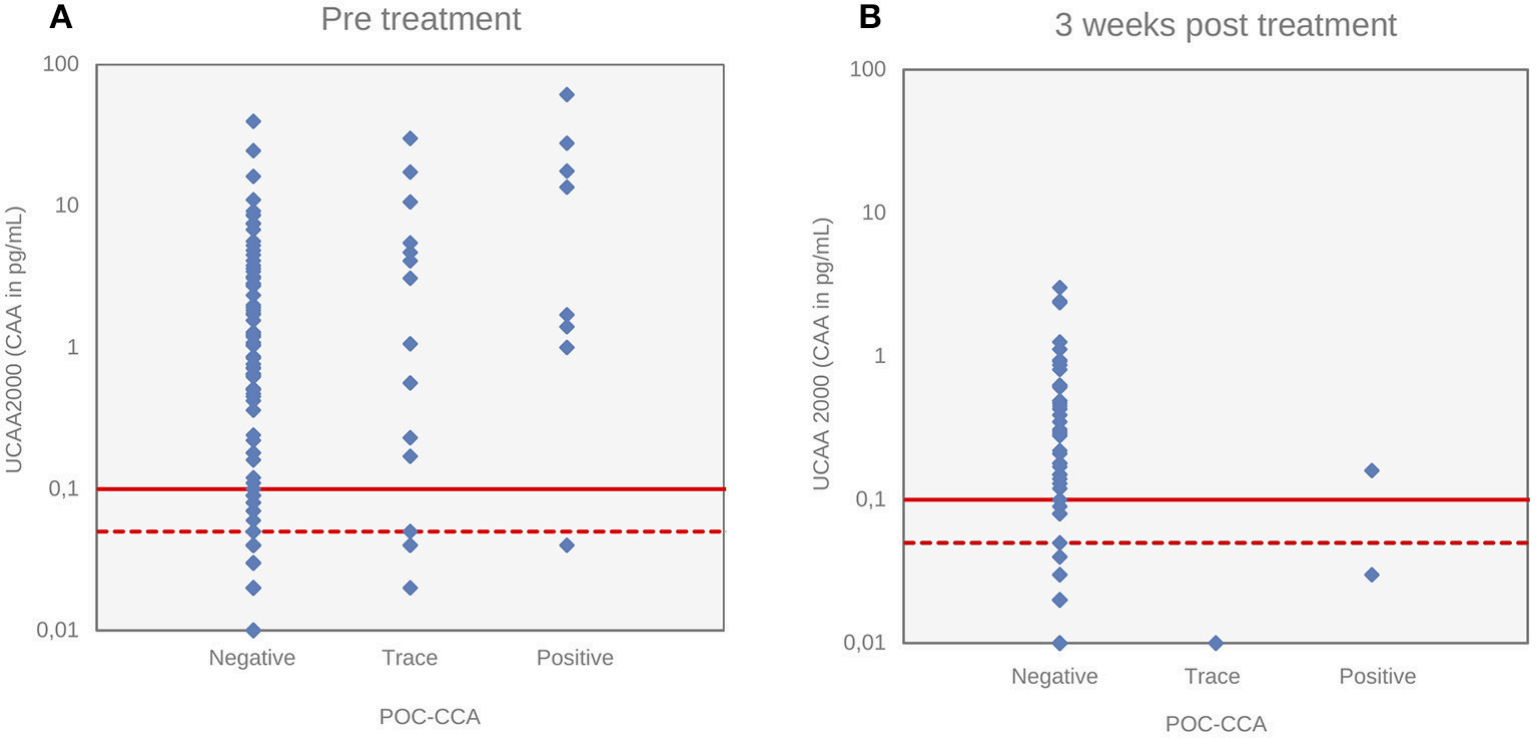
Correlations of *S. mansoni* urine CAA levels with POC-CCA intensity scores. Correlations of CAA levels (pg/mL) determined by the UCP-LF CAA assay (UCAA 2000) with POC-CCA intensity scores at baseline **(A)** and 3 weeks after drug treatment **(B)**. The solid red lines represent the high-specificity cut-off levels, while the dotted red lines indicate lower specificity levels for the UCP-LF CAA assay. Samples that have concentrations in the region between the dotted line and the solid line are classified as “indecisive”.

Figure 4 depicts the correlations between the CAA concentrations and POC-CCA intensity scores at baseline (panel A, Spearman's rho = 0.24, *p* < 0.001) and at 3 weeks after drug administration (panel B, Spearman's rho = −0.043, *p* = 0.63). Panel A shows that eleven “trace” signals of the twenty POC-CCA tests were confirmed positive by the UCP-LF CAA test.

## Discussion

The current study highlights the potential of serum as well as urine samples for a CAA diagnostic approach to accurately determine the *S. mansoni* infections using the ultra-sensitive UCP-LF assay, as evaluated for the first time in a low endemic area in Brazil. The observed prevalence was significantly higher with the antigen approach: increasing from 1.6% by Kato-Katz to about 31% by the UCP-LF CAA assays; an almost twenty-fold increase. The UCAA2000 and SCAA500 assays identified additional 78 and 80 positive cases with respect to Kato-Katz technique, respectively. The genus specific UCP-LF CAA assays have been well evaluated in various endemic settings and previous studies (46–48) showing considerably more positive cases both for the different intestinal as well as urinary *Schistosoma* species. The strength and uniqueness of the current study is the evaluation of the CAA levels both in serum and urine on 2 time-points relatively shortly after treatment.

Despite of the guidance of Brazilian Schistosomiasis Control Program (two slides), the Kato-Katz technique was performed on three slides from one stool. However, the method was only 3.6% sensitive against the ultrasensitive UCP-LF CAA assays for *S. mansoni* diagnosis, thus providing further evidence that the widely used Kato-Katz technique is insensitive in low-endemic areas (9, 58). The introduction of the UCP reporter technology was a substantial factor for the increase in sensitivity (59). The applied 400 nm Y_2_O_2_S:Yb^3+^, Er^3+^ luminescent (phosphorescence) reporter particles compared to other conventionally applied (fluorescent) labels include advantages such as high sensitivity (no auto fluorescence from other assay components), long shelf life and a permanent record (no fading). Furthermore, the addition of a concentration step of the samples after extraction with TCA allowed the use of larger volumes of urine (2 mL) and serum (0.5 mL) in the UCP-LF assay. It implied a significant increase in its analytical sensitivity, as indicated in laboratory studies by Corstjens et al. (29, 30). Clearly better strategic decisions will be made with more sensitive diagnostics (60).

In the current study, the high cut-off threshold that was chosen according to the protocol outlined by Corstjens et al. (29) turned out to be quite conservative and contributed to the nearly 100% specificity. Negative quality controls (QC) and standard series indicated that in fact a slightly lower cut-off was allowed. However, in areas with low prevalence and/or low parasitic load, settings that are moving toward elimination, assay accuracy, and particularly specificity is very important. Therefore, studies with the UCP-LF CAA assay have been indicated the application of two cut-off values, allowing an analysis with lower or greater (100%) specificity, depending on the hypothesis or the purpose of the study, which implied the definition of a group that was designated “potentially positive” in early studies (40, 61) and more recently called “indecisive” (29, 35, 47). The detection by highly specific monoclonal antibodies (62), the extraction of CAA by TCA sample pre-treatment and the antigen uniqueness (63) are the main arguments for the high specificity of the UCP-LF CAA assay used in this study.

The commercially available POC-CCA urine cassette test showed sensitivity higher to Kato-Katz technique (29.1 vs. 3.6%, including trace results as positive). Our data corroborate findings from others in different African settings (34) and more recent findings in a Brazilian low-endemicity area (38) that have shown that the POC-CCA is capable of detecting many additional positive cases compared to Kato-Katz thick smears. Advantages include direct savings in terms of the costs of testing and treatment delivery as well as considerable savings in time for collection and processing of samples (urine instead of stool), and compliance of patients (34, 64). On the other hand, in this low endemic area, the POC-CCA test was much less sensitive than the UCP-LF CAA assays. Indeed, 62 individuals detected by the UCAA2000- assay were negatives by POC-CCA analysis, and therefore would be incorrectly found negative by this the latter test. Moreover, the common appearance of “trace” signals (very light lines) was raised previously due to its reader subjectivity and the need for reading standardization has been discussed (34, 38). Recently, a study was performed using image analysis to quantify the color of the lines in the strip of the cassette (65). Also, a study in a Brazilian population with low parasite burden in an endemic area was designed to clarify the interpretation of the concept of trace showed that after a 10-fold concentration of urine samples by lyophilization, the trace became positive in parasitological positive cases, but remained as trace in parasitological negative cases, indicating trace readings could not be promptly defined as positive or negative (37). Using the UCP-CAA assay as a “confirmatory” test, according Colley et al. (16), we noticed that eleven POC-CCA “trace” signals out of twenty were confirmed positive by UCAA2000-. Moreover, 18 POC-CCA positive but egg-negative cases were also confirmed positive. Evaluation the POC-CCA against a comparative reference of urine and serum CAA, PCR and KK results, showed a sensitivity and specificity of 29 and 89% if traces were considered positive, and 11 and 97%, respectively, if traces were considered negative.

The PCR showed sensitivity significantly higher to Kato-Katz technique (34.5 vs. 3.6%), as expected and demonstrated elsewhere (18, 21). Also the molecular method was more sensitive than the POC-CCA test, corroborating with findings from other authors (25, 66). Noteworthy, six out of the 19 PCR positive individuals were not detected by UCP-LF CAA assays. However, PCR was less sensitive than the UCP-LF CAA assays. This can be explained in part by the fact that the fecal aliquot used may not contain the minimum amount of parasite egg required to obtain DNA in the extraction stage, since they are samples from individuals with low parasitic load. The number of excreted eggs is often low and shows high day-to-day fluctuation (67), with consequent unequal distribution of these in stool samples. In addition, although cost reduction can be achieved by methodological modifications, such as the improvement of DNA extraction methods (68), the PCR remains an expensive method. In fact, a limitation of our study and approaches to estimate sensitivity and specificity is that the PCR was not performed in all samples.

The antibody detection is considered highly sensitive and it has been recommended as a complementary tool for the schistosomiasis diagnosis for individuals with low infection burden, which are usually hard to detect by parasitological methods (11, 13). However, our results show that one out of the four egg-positive cases and 51 (53.1%) of the CAA positives were antibody negative. This was reported previously in a Chinese setting characterized by a very low prevalence of *S. japonicum* (47) and could be explained by an effect of immune down-regulation in chronic schistosome infections (69), any immune incompetence or it could be due to the test itself. The performances of antibody assays do vary a lot (70, 71). Anyway, this implies that if we using the SWAP-ELISA as a first-line screening tool, more than half of the active infections would be missed, thereby allowing these patients to go untreated and continue to contribute to transmission. The low specificity of the ELISA (73%) would also lead to the conclusion that many individuals would be positive, which actually did not have active infections.

Categorizing the urine and serum CAA and egg-DNA positivity by age, the highest positivities were found in older people (nearly of the 30–39 olds 50% were positive by CAA). However, also many people below 20 years old tested positive. Although most studies are based on schoolchildren populations, a study conducted in Zambia using Kato-Katz, POC-CCA, and PCR methods similarly revealed that there are high prevalence rates of *S. mansoni* infections in adults (66), while a study in a low endemic area in China showed that prevalences were highest in even older age groups with a maximum in 40–49 year old people (47). This is an important observation from the strategic point of view for decision-making in the Control Programs, because since the control approach for areas with prevalence levels between 10 and 50% is preferentially aimed at the treatment of only the schoolchildren (72, 73), many of these positive patients would remain untreated and continue to contribute to the transmission of the disease. Moreover, we found children before their sixth birthday infected, which reinforce that preschool-aged children are at risk of schistosomiasis as described elsewhere (50, 57), and the treatment these little children also is recommended by WHO (74) and increasingly encouraged (75).

We found 35 (27.3%) and 11 (8.9%) positive patients by the UCAA2000- and the SCAA500- at 6-weeks post-treatment, respectively. It would be very interesting to design further research with a good protocol which assists elucidating these findings, but at least four points need to be discussed. First, 6 weeks should be enough for antigen clearance (51, 76), in particular in these rather low concentrations, so the presence of CAA is still indicative for the presence of remaining worms, although at a very low load. Second, a study showed that cure rates against *S. mansoni*, determined 1 month after treatment, ranged from 52 to 92% (77). In addition, a systematic review revealed a global cure rate of 71.3% of praziquantel against *S. haematobium* and *S. mansoni* (78). Thus, these studies, among others based on both the detection of circulating antigens (31, 32) and by PCR (79), have already demonstrated that the effectivity of praziquantel is much lower indicating that many worms may survive. Also, it is known that worms may be affected but will recover from the PZQ attack (16, 29). Third, the killing of the worms is dependent on an active immune response, so in people with some immunosuppression the worms may relatively easily survive (80–82). Finally, when there are adult worms, and transmission is going on, there will also be young worms, which are not affected by PZQ (83). They will grow to adult worms over a period of weeks and will start to excrete higher CAA levels.

Despite the considerable number of positive patients by UCP-LF CAA after treatment, it was found that CAA concentrations in these individuals were quite low. Our findings showed a significant reduction in CAA concentrations 3 and 6 weeks after treatment, which is in accordance with the findings of Corstjens et al. (29) that showed a decrease in urine CAA concentrations 2 months after administration of praziquantel in *S. haematobium* infected individuals. These findings confirm that determination of CAA levels immediately before and shortly after drug administration will be a better indicator for monitoring drug efficiency than monitoring egg production.

Serum CAA concentrations were found to be significantly higher than urine levels, as has generally been observed in previous studies employing CAA-ELISA (43, 84) or more recently using UCP-LF CAA assay (47). In contrast to a previous study in *S. mekongi* infected individuals (35) we obtained only a low correlation between CAA concentrations and POC-CCA intensity scores, which could be explained by the very low parasite burden in this setting, resulting in relatively low POC-CCA signals. Consequently, the number of positive individuals detected by the much more sensitive UCP-LF CAA test was significantly higher than by POC-CCA.

Although the absence of a true gold standard could be considered as a limitation, the combined reference standard method has been used before with the discussed considerations and limitations (46–48) and has actually been recommended for such a situation by a WHO/TDR (the Special Programme for Research and Training in Tropical Diseases) working group (85). We constructed a combined reference standard composite by Kato-Katz and/or PCR and/or POC-CCA results for indication of the performances of the UCP-LF CAA assays and of the SWAP-ELISA; posteriorly, a comparative reference composited by Kato-Katz and/or PCR and/or CAA results was done but only for comparative reasons in an attempt to indicate of the performances of all diagnostic tests. This last one allowed the results to be compared to other similar studies which applied the same analysis approach, which warrants its importance. It is worth of note that sensitivities and specificities parameters from the UCP-LF CAA assays and mainly from the SWAP-ELISA were similar when analyzed against both combined references. The Bayesian latent class analyses (LCA) is an alternative statistical approach which was applied in a different study (34, 49). In such a statistically more elaborate approach with UCP-LF CAA not being part of the reference, the results would most probably be very similar as observed by Knopp et al. (48) that applied both methods. A further option for future studies would be to use a more sensitive parasitological test as (approximate) gold standard for active infection, such as Helmintex (86). This alternative for egg microscopy would improve diagnosis of infections with low burdens and was recently optimized (e.g., to decrease the time for completing the examination) (87). However, the method is still very labor intensive and time-consuming for field applications (88). A rapid and cost-effective diagnostic test capable of detecting very low numbers of schistosome worms is urgently needed when moving to the elimination stage of schistosomiasis control programs (64). PCR and detection of circulating antigens presents significant differences from diagnosis by traditional stool microscopy and serology methods. While serology is an indirect technique not distinguishing between active and past infection, the other approaches are direct and therefore applicable, depending on sensitivity, to indicate infection status and treatment outcomes. Detection of the rapidly cleared worm regurgitated circulating antigens CAA and CCA may be preferred also given cost issues and lower sensitivity due the dependence on the presence of egg-derived DNA for most of the current available PCR methods. While the POC-CCA is commercially available at relatively low cost ($1–$1.5 per test) a drawback of the UCP-LF CAA test is that it is currently still a laboratory based test, with its application limited to collaborative research projects.

In summary, the UCP-LF CAA assay was employed for first time to urine and serum samples from *S. mansoni*-infected individuals from Brazil before and after praziquantel treatment. Extending the existing diagnostic arsenal is one of the essential requirements for successful disease control. The significantly higher prevalences of active *Schistosoma* infections detected by UCP-LF CAA assays shown in the current study, its potential utility for evaluating cure rates after treatment, and the fact this test can be performed on urine samples (less invasive collecting) taken together makes of the UCP-LF CAA assay a sensitive, robust and promising diagnostic approach, especially in low-endemicity settings.

## Author Contributions

FB, GvD, MS, ED, and MP: conceived and designed the experiments; MS, GvD, CdD, MP, RP, and JP: performed the experiments; MS, GvD, CdD, PC, FB: analyzed the data; FB, GvD, CdD, PC, JP, RP, ED: contributed reagents, materials, analysis tools; MS: wrote the first draft of the manuscript; MS, GvD, PC, FB, and ED: wrote sections and revising it critically. All the authors approved the final version of the manuscript.

### Conflict of Interest Statement

The authors declare that the research was conducted in the absence of any commercial or financial relationships that could be construed as a potential conflict of interest.
